# Synchrotron powder study of Na_3_V(PO_3_)_3_N

**DOI:** 10.1107/S1600536813012427

**Published:** 2013-05-11

**Authors:** Minwoong Kim, Seung-Joo Kim

**Affiliations:** a Department of Chemistry, Division of Energy Systems Research, Ajou University, Suwon 443-749, Republic of Korea

## Abstract

Polycrystalline tris­odium vanadium(III) nitridotriphosphate, Na_3_V(PO_3_)_3_N, was prepared by thermal nitridation of a mixture of NaPO_3_ and V_2_O_5_. The title compound is isotypic with Na_3_Al(PO_3_)_3_N. In the crystal, the P-atom and the three O-atom sites are on general positions, whereas the Na-, V- and N-atom sites are located on threefold rotation axes. The P atom is coordinated by three O atoms and one N atom in form of a slightly distorted tetra­hedron. Three PO_3_N tetra­hedra build up a nitridotriphosphate group, (PO_3_)_3_N, by sharing a common N atom. The V atom is coordinated by six O atoms in form of a slightly distorted octa­hedron. The Na^+^ ions occupy three crystallographically distinct sites. One Na^+^ ion is situated in an irregular polyhedral coordination environment composed of six O atoms and one N atom, while the other two Na^+^ cations are surrounded by six and nine O atoms, respectively.

## Related literature

For structure determination of the isotypic Na_3_Al(PO_3_)_3_N, see: Conanec *et al.* (1994 ▶). For the preparation of various related materials, *A*_3_ *B*(PO_3_)_3_N (*A* = Na, K; *B* = Al, Ga, Cr, Mn, Fe) and *A*_2_ *B*_2_(PO_3_)_3_N (*A* = Na; *B* = Mg, Mn, Fe, Co), see: Conanec *et al.* (1996 ▶); Feldmann (1987**a* ▶,b* ▶). For studies focused on the ionic conductivity of Na_2_Mg_2_(PO_3_)_3_N, see: Lee *et al.* (2012 ▶). For a review of structural features of metal nitridophosphate compounds, see: Marchand & Laurent (1991 ▶); Marchand *et al.* (2000 ▶). For bond-valence-sum calculations, see: Brese & O’Keeffe (1991 ▶). For comparison of bond lengths in related structures, see: Conanec *et al.* (1994 ▶); Jacobs & Nymwegen (1997 ▶); Lee *et al.* (2012 ▶); Shannon (1976 ▶); Zatovsky (2010 ▶).

## Experimental

### 

#### Crystal data

Na_3_V(PO_3_)_3_N
*M* *_r_* = 370.83
Cubic, 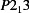

*a* = 9.44783 (5) Å
*V* = 843.33 (1) Å^3^
*Z* = 4
Synchrotron radiation, λ = 1.547400 Å
*T* = 298 K
Flat sheet, 20 × 20 mm

#### Data collection

Pohang Light Source 9B HRPD Beamline diffractometer
Specimen mounting: packed powder pellet
Data collection mode: reflection
Scan method: step
2θ_min_ = 10.060°, 2θ_max_ = 130.500°, 2θ_step_ = 0.010°

#### Refinement

*R*_p_ = 0.091
*R*_wp_ = 0.119
*R*_exp_ = 0.075
*R*_Bragg_ = 0.056
χ^2^ = 2.519
12045 data points
288 parameters

### 

Data collection: local software at 9B HRPD beamline; cell refinement: *DICVOL* (Boultif & Louër, 2004 ▶); data reduction: local software at 9B HRPD beamline; method used to solve structure: coordinates taken from an isotypic compound; program(s) used to refine structure: *FULLPROF* (Rodriguez-Carvajal, 2001 ▶); molecular graphics: *DIAMOND* (Brandenburg, 1999 ▶); software used to prepare material for publication: *FULLPROF*.

## Supplementary Material

Crystal structure: contains datablock(s) global, I. DOI: 10.1107/S1600536813012427/wm2731sup1.cif

Rietveld powder data: contains datablock(s) I. DOI: 10.1107/S1600536813012427/wm2731Isup2.rtv

Additional supplementary materials:  crystallographic information; 3D view; checkCIF report

## Figures and Tables

**Fig. 1.**
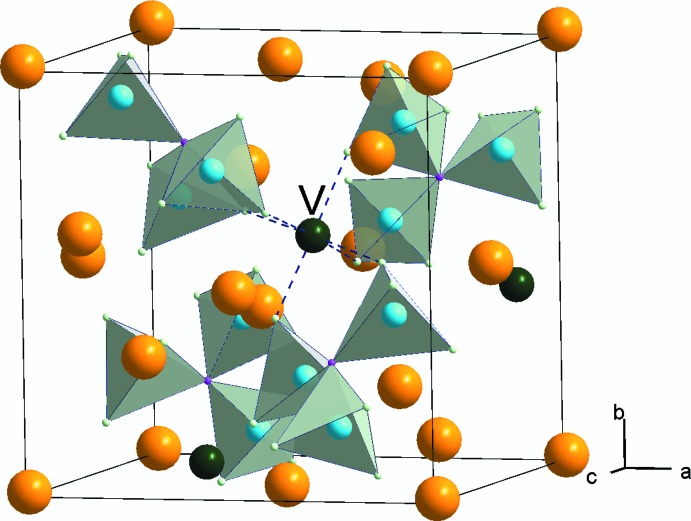
The crystal structure of Na3V(PO3)3N. PO4 tetrahedra are shown with gray shading; Na atoms are orange-colored spheres and V atoms are dark green spheres.

**Fig. 2.**
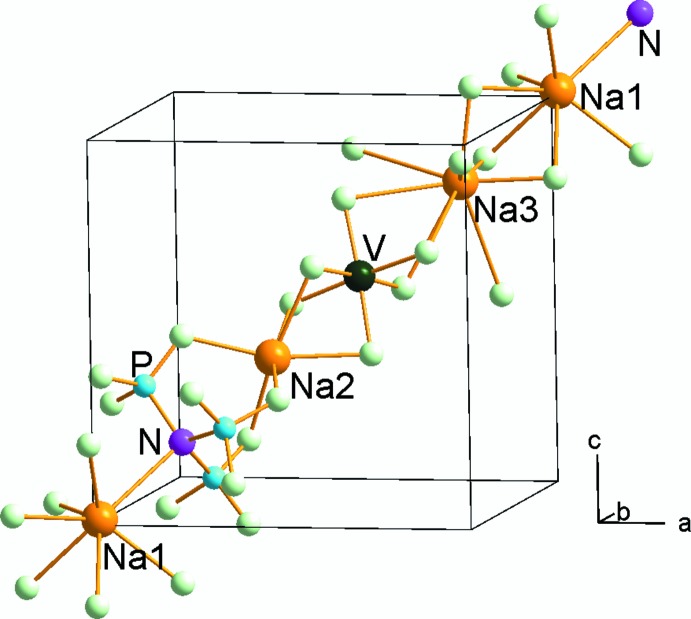
The local environments of Na, V and N atoms arranged along [111] in the structure of Na3V(PO3)3N.

**Fig. 3.**
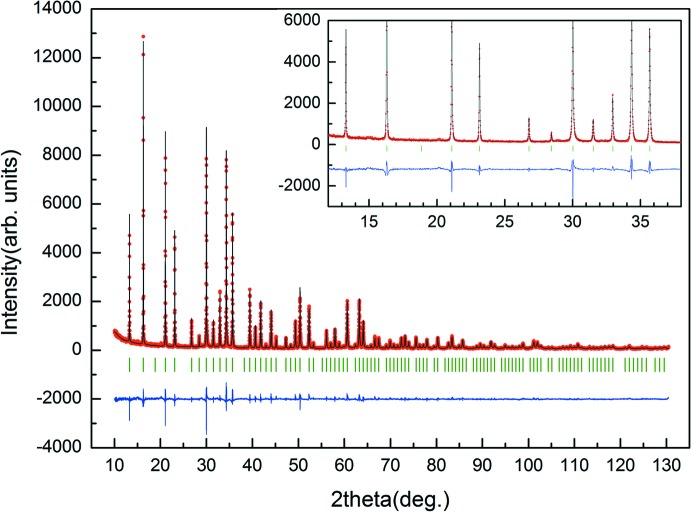
Rietveld refinement plot of Na3V(PO3)3N based on synchrotron X-ray powder diffraction data. The inset shows an enlarged section of the diffraction pattern.

## supplementary crystallographic information

### Comment

There has been growing interest in the area of mixed anionic systems for exploration of new functional materials. Synthesis in the area of mixed anionic systems allows for the tuning of numerous properties, including energy storage, photocatalytic, and dielectric properties. More than two decades ago, the synthesis of several isotypic compounds with the chemical formulae of *A*_3_*B*(PO_3_)_3_N (*A* = Na, K; *B* = Al, Ga, Cr, Mn, Fe) and *A*_2_*B*_2_(PO_3_)_3_N (*A* = Na; *B* = Mg, Mn, Fe, Co) by versatile nitridation reactions have been reported (Conanec *et al.*, 1996; Feldmann, 1987**a*,b*; Marchand & Laurent, 1991, Marchand *et al.*, 2000) The crystal structure of Na_3_Al(PO_3_)_3_N has been determined by X-ray diffraction from a single-crystal (Conanec *et al.*, 1994). For the other compounds, however, no information except their unit cell parameters are known. In this work, we report the synthesis of the new nitridophosphate compound, Na_3_V(PO_3_)_3_N, and its crystal structure refined on baisi of the Rietveld method from synchrotron powder X-ray diffraction data.

The crystal structure of Na_3_V(PO_3_)_3_N is isotypic with that of Na_3_Al(PO_3_)_3_N (Conanec *et al.*, 1994). The lattice parameter of Na_3_V(PO_3_)_3_N (*a* = 9.44783 (5) Å) is slighty larger than that of Na_3_Al(PO_3_)_3_N (*a* = 9.274 (1) Å), which is attributed to the different sizes of the V(III) and Al(III) ions. In this structure, the P and the three O atoms lie on general positions (12*b*) while the other atoms lie on special positions related to threefold rotation axes (4*a*).

The P atom is coordinated by three O atoms and one N atom to form a PO_3_N tetrahedron. The (PO_3_)_3_N entity is formed by three PO_3_N tetrahedra sharing the corner occupied by the nitrogen atom (Fig. 1). The range of P—O bond lengths (1.538 (6) - 1.541 (6) Å) in Na_3_V(PO_3_)_3_N is close to that found in compositionally related compounds such as Na_3_Al(PO_3_)_3_N (~1.50 - 1.53 Å); Na_2_Mg_2_(PO_3_)_3_N (~1.53 - 1.55 Å); Na_3_V_2_(PO_4_)_3_ (~1.52 1.54 Å) (Conanec *et al.*, 1994; Lee *et al.*, 2012; Zatovsky, 2010). The P—N bond length (1.738 (7) Å) is similar to those observed for tricoordinating nitrogen atoms in nitrido-compounds such as K_3_P_6_N_11_ (1.71 Å) (Jacobs & Nymwegen, 1997). Na, V and N atoms are arranged along the [111] direction in the sequence of Na2—V—Na3—Na1—N—Na2—V-··· (Fig. 2). The V atom is connected to six oxygen atoms located at the vertices of PO_3_N tetrahedra, forming a slightly distorted octahedron. The average V—O distance is 2.005 (11) Å which is close to the sum of the ionic radii (2.02 Å) of V^3+^ and O^2-^ (Shannon, 1976). The Na atoms occupy three crystallographically distinct sites. Na1 is coordinated to six O atoms (mean Na—O is 2.58 Å) and one N atom (Na—N is 2.947 (7) Å) to form an irregular NaO_6_N polyhedron. Na2 and Na3 are 6- and 9-coordinated, respectively, within distorted polyhedra elongated along the threefold axis. The bond valence sums (Brese & O'Keeffe, 1991) calculated from the bond lengths (valence units; Na1: 0.80, Na2: 1.29, Na3: 0.96, V: 2.96, P: 4.61, O1: 1.90, O2: 1.92, O3: 1.85, N: 2.80) are close to the expected valence states of respective atoms.

### Experimental

An appropriate amount of NaPO_3_ and V_2_O_5_ was mixed thoroughly in an agate mortar and placed in an alumina crucible. The mixture was initially heated at 523 K for 6 h. The product was reground and heated again at 973 K for 8 h and furnace-cooled to room temperature. All the heat treatments were carried out in continuous flowing anhydrous ammonia gas (flow rate = 30 ml/min) in a tube furnace. The resultant powder sample was characterized by synchrotron X-ray diffraction (sXRD). The measurement was performed on beamline 9B-HRPD at Pohang Accelerator Laboratory, Pohang, Korea. The incident X-rays were vertically collimated by a mirror, and monochromated to the wavelength of 1.5474 Å by a double-crystal Si (111) monochromator. The datasets were collected in the range of 10°≤ 2θ≤ 130° with a step size of 0.01° (2θ range).

### Refinement

Reflections were indexed using *DICVOL* (Boultif & Louër, 2004). The cubic symmetry was obviously obtained from sXRD data. Any additional peaks were not detected. The figures of merit were *M*(20) = 277.6 (19), F(20) = 378 (3). Systematic absences, *h* = 2*n* + 1 for *h*00 observed in the intensity data, suggested the space group *P*2_1_3. As an initial model for the Rietveld refinements, the structural parameters of Na_3_Al(PO_3_)N from single crystal data (Conanec *et al.*, 1994) were used. Refinements of structural parameters were carried out using the Fullprof program package (Rodriguez-Carvajal, 2001). The shape of the diffraction peaks was modelled with the Thompson-Cox-Hastings pseudo-Voigt function. A manual background correction was used in the refinements; preferred orientation and absorption effects were not considered. In the final refinement run, the following parameters were refined: zero shift, peak width/shape/asymmetry, scale factor, and crystal structure parameters (lattice parameter, atomic positions, isotropic atomic displacement parameters). The final refinement plot is shown in Fig. 3.

### Crystal data

**Table:**

Na_3_V(PO_3_)_3_N	*D*_x_ = 2.92 Mg m^−^^3^
*M**_r_* = 370.83	Synchrotron radiation, λ = 1.547400 Å
Cubic, *P*2_1_3	*T* = 298 K
Hall symbol: P 2ac 2ab 3	Particle morphology: powder
*a* = 9.44783 (5) Å	green
*V* = 843.33 (1) Å^3^	flat sheet, 20 × 20 mm
*Z* = 4	

### Data collection

**Table:**

Pohang Light Source 9B HRPD Beamline diffractometer	Data collection mode: reflection
Radiation source: synchrotron	Scan method: step
Si 111 monochromator	2θ_min_ = 10.060°, 2θ_max_ = 130.500°, 2θ_step_ = 0.010°
Specimen mounting: packed powder pellet	

### Refinement

**Table:**

*R*_p_ = 0.091	12045 data points
*R*_wp_ = 0.119	35 parameters
*R*_exp_ = 0.075	0 restraints
*R*_Bragg_ = 0.056	(Δ/σ)_max_ = 0.02
χ^2^ = 2.519	

### Fractional atomic coordinates and isotropic or equivalent isotropic displacement parameters (Å^2^)

**Table:**

	*x*	*y*	*z*	*U*_iso_*/*U*_eq_	
P1	0.3326 (3)	0.0844 (3)	0.2446 (3)	0.0143 (4)*	
V1	0.08073 (17)	−0.08073 (17)	0.41927 (17)	0.0144 (6)*	
Na1	0.0136 (3)	0.0136 (3)	0.0136 (3)	0.0278 (18)*	
Na2	0.3913 (4)	0.3913 (4)	0.3913 (4)	0.0171 (18)*	
Na3	0.6989 (5)	0.1989 (5)	0.3011 (5)	0.0310 (19)*	
O1	0.2722 (6)	−0.0265 (6)	0.3479 (5)	0.0130 (14)*	
O2	0.3727 (5)	0.0002 (6)	0.1109 (5)	0.0073 (15)*	
O3	0.4543 (6)	0.1700 (6)	0.3106 (6)	0.0160 (18)*	
N1	0.1937 (7)	0.1937 (7)	0.1937 (7)	0.012 (3)*	

### Geometric parameters (Å, º)

**Table:**

P1—O3	1.538 (6)	Na1—N1	2.947 (7)
P1—O2	1.540 (6)	Na2—O3^viii^	2.304 (7)
P1—O1	1.541 (6)	Na2—O3^v^	2.304 (7)
P1—N1	1.738 (7)	Na2—O3	2.304 (7)
V1—O1^i^	1.997 (6)	Na2—O2^ix^	2.456 (6)
V1—O1^ii^	1.997 (6)	Na2—O2^x^	2.456 (6)
V1—O1	1.997 (6)	Na2—O2^xi^	2.456 (6)
V1—O2^iii^	2.013 (5)	Na3—O3	2.329 (7)
V1—O2^iv^	2.013 (5)	Na3—O3^x^	2.329 (7)
V1—O2^v^	2.013 (5)	Na3—O3^xii^	2.329 (7)
Na1—O1^vi^	2.561 (6)	Na3—O1^xiii^	2.964 (7)
Na1—O1^vii^	2.561 (6)	Na3—O1^xiv^	2.964 (7)
Na1—O1^i^	2.561 (6)	Na3—O1^xi^	2.964 (7)
Na1—O3^vii^	2.604 (6)	N1—P1^viii^	1.738 (7)
Na1—O3^i^	2.604 (6)	N1—P1^v^	1.738 (7)
Na1—O3^vi^	2.604 (6)		
			
O3—P1—O2	114.9 (6)	O1—V1—O2^iii^	90.5 (4)
O3—P1—O1	112.2 (6)	O1^i^—V1—O2^iv^	92.2 (4)
O2—P1—O1	105.0 (5)	O1^ii^—V1—O2^iv^	90.5 (4)
O3—P1—N1	111.4 (6)	O1—V1—O2^iv^	176.8 (5)
O2—P1—N1	105.4 (6)	O2^iii^—V1—O2^iv^	86.5 (3)
O1—P1—N1	107.4 (6)	O1^i^—V1—O2^v^	90.5 (4)
O1^i^—V1—O1^ii^	90.8 (4)	O1^ii^—V1—O2^v^	176.8 (5)
O1^i^—V1—O1	90.8 (4)	O1—V1—O2^v^	92.2 (4)
O1^ii^—V1—O1	90.8 (4)	O2^iii^—V1—O2^v^	86.5 (3)
O1^i^—V1—O2^iii^	176.8 (5)	O2^iv^—V1—O2^v^	86.5 (3)
O1^ii^—V1—O2^iii^	92.2 (4)		

Symmetry codes: (i) −*y*, *z*−1/2, −*x*+1/2; (ii) −*z*+1/2, −*x*, *y*+1/2; (iii) −*x*+1/2, −*y*, *z*+1/2; (iv) −*z*, *x*−1/2, −*y*+1/2; (v) *y*, *z*, *x*; (vi) −*x*+1/2, −*y*, *z*−1/2; (vii) *z*−1/2, −*x*+1/2, −*y*; (viii) *z*, *x*, *y*; (ix) −*z*+1/2, −*x*+1, *y*+1/2; (x) *y*+1/2, −*z*+1/2, −*x*+1; (xi) −*x*+1, *y*+1/2, −*z*+1/2; (xii) −*z*+1, *x*−1/2, −*y*+1/2; (xiii) *y*+1, *z*, *x*; (xiv) *z*+1/2, −*x*+1/2, −*y*.
